# Impact of derived global weather data on simulated crop yields

**DOI:** 10.1111/gcb.12302

**Published:** 2013-09-24

**Authors:** Justin van Wart, Patricio Grassini, Kenneth G Cassman

**Affiliations:** Department of Agronomy and Horticulture, University of Nebraska-LincolnLincoln, NE, 68583-0915, USA

**Keywords:** crop model, maize, rice, weather data, wheat, yield potential

## Abstract

Crop simulation models can be used to estimate impact of current and future climates on crop yields and food security, but require long-term historical daily weather data to obtain robust simulations. In many regions where crops are grown, daily weather data are not available. Alternatively, gridded weather databases (GWD) with complete terrestrial coverage are available, typically derived from: (i) global circulation computer models; (ii) interpolated weather station data; or (iii) remotely sensed surface data from satellites. The present study's objective is to evaluate capacity of GWDs to simulate crop yield potential (Yp) or water-limited yield potential (Yw), which can serve as benchmarks to assess impact of climate change scenarios on crop productivity and land use change. Three GWDs (CRU, NCEP/DOE, and NASA POWER data) were evaluated for their ability to simulate Yp and Yw of rice in China, USA maize, and wheat in Germany. Simulations of Yp and Yw based on recorded daily data from well-maintained weather stations were taken as the control weather data (CWD). Agreement between simulations of Yp or Yw based on CWD and those based on GWD was poor with the latter having strong bias and large root mean square errors (RMSEs) that were 26–72% of absolute mean yield across locations and years. In contrast, simulated Yp or Yw using observed daily weather data from stations in the NOAA database combined with solar radiation from the NASA-POWER database were in much better agreement with Yp and Yw simulated with CWD (i.e. little bias and an RMSE of 12–19% of the absolute mean). We conclude that results from studies that rely on GWD to simulate agricultural productivity in current and future climates are highly uncertain. An alternative approach would impose a climate scenario on location-specific observed daily weather databases combined with an appropriate upscaling method.

## Introduction

Anthropogenic greenhouse gas emissions are likely to modify climate in coming decades (Allen *et al.*, 2000; Oreskes, 2004), and there is increasing concern about impact of climate change on food security (IPCC, 2007; Schmidhuber & Tubiello, 2007). A key question is how future climates will influence capacity to produce adequate food supply at regional to national and global scales. To date, most studies examining global impacts of climate change on crop yields have been based on derived, gridded weather databases (GWDs) that provide complete coverage of earth's terrestrial surface (e.g. Fischer *et al.*, 2002; Foley *et al.*, 2005; Licker *et al.*, 2010; Ciais *et al.*, 2011). At issue is how well such GWDs perform in estimating food production potential in today's climate, which is the central focus of our study.

Establishing research plots in every geographic area of interest to analyze effects of climate on crop production is difficult and cost prohibitive. For this reason agronomists turn to crop simulation models, which capture major interactions among crop genotype, environment, and management. Most previous studies that utilized crop simulation models to evaluate impact of climate change on crop yields have assumed (implicitly or explicitly) that crops were grown with optimal management (Rosenzweig & Parry, 1994; Fischer *et al.*, 2002; Bondeau *et al.*, 2007; Tubiello *et al.*, 2007). This assumption is made because currently available simulation models do not account for all of the interacting constraints that limit crop growth and yield in farmer's fields such as deficient or imbalanced supply of 16 essential nutrients, inadequate or excessive water supply, and yield losses from insect pests, weeds, and diseases. In addition, crop yields can be decreased by imperfect field management that leads to unintended suboptimal plant population or uneven plant stands, effects not accounted for in some crop models. In contrast, under optimal conditions, and when grown with irrigation, crop yield potential (Yp) is determined solely by plant population and solar radiation and temperature during the period from planting to maturity. Evans (1993) defined Yp as the yield of an adapted crop cultivar grown under conditions in which nutrients, pests, and diseases are nonlimiting. When crops are grown without irrigation (i.e. rainfed conditions), a water-limited yield potential (Yw) is determined by the same factors that influence Yp, but also by water supply (soil water at planting plus in-season precipitation) and soil characteristics that affect the plant-available water supply. Simulation of yield potential is relatively straightforward because dry matter accumulation relies solely on the balance between photosynthesis and respiration, and seed yield is determined by partitioning of total dry matter between seed and vegetative organs. All three of these processes are relatively well understood such that underpinning mechanisms can be described in a set of mathematical formulas that comprise the core of crop simulation models. Future crop yields are expected to be producing much nearer yield potential due to increased food demand but limited land and water resources for expansion of agriculture (Godfray *et al.*, 2010). Thus, within the context of climate change and a time horizon of several decades, a focus on yield potential provides a robust proxy for future food production.

Yield potential can be simulated using site-specific, observed weather data or gridded weather data. Gridded weather data are distributed uniformly over space within a spatial grid cell. Values within a cell are typically derived by interpolating site-specific weather data based on coordinates of the sites within the grid and in nearest-neighbor grids, their distance from each other, elevation, and other variables (Hutchinson, 1995; Boer *et al.*, 2001). Gridded weather data have the advantage of full geospatial coverage, but they are derived, rather than observed. Studies that have used gridded weather data to simulate Yp or Yw for a grid are rarely validated against Yp or Yw estimated using actual weather station data from a location within the same grid (Fischer *et al.*, 2002; Foley *et al.*, 2005; Lobell *et al.*, 2008).

More than 30 weather data sources have been used in agricultural research, but only a few of these have been used for global-scale analysis of simulated yields (Ramirez-Villegas & Challinor, 2012). The main differences among sources of those weather databases used to simulate Yp and Yw include: (i) observed site-based vs. interpolated gridded data; (ii) temporal resolution (daily vs. monthly); and (iii) spatial resolution (among gridded databases) (Table 1). Several studies have compared simulated yields using observed, site-specific data with simulations made using gridded or modeled weather data (Mearns *et al.*, 2001; Baron *et al.*, 2005; van Bussel *et al.*, 2011), but these studies only focus on a single source of gridded weather data without considering other databases with different spatial and temporal attributes.

**Table 1:** Classification of global weather databases and examples of published studies using these databases to understand current and future agricultural productivity. Weather databases used in the present study have been underlined

Classification	Source	Time step	Reference and time interval	Geospatial coverage	Reported variables^*^	Examples
Point-based data	Weather stations	Daily	HPRCC^†^, CMA^‡^, DWD^§^ (1983–2010)	Regional	*T*_min_, *T*_max_, precip, wind speed, Tdew Temp, RH, vapor pressure, radiation	Sinclair & Rawlins (1993), Wang & Connor (1996), Peng *et al*. (2004), Grassini *et al*. (2009), Cassman *et al*. (2010)
			NOAA¶(1900–2010)	Global	*T*_min_, *T*_max_, precip, Tdew, wind speed, RH, vapor pressure	
Gridded data	Interpolated and generated based on data from weather stations, satellites, ocean buoys, etc.	Daily	NCEP/DOE Reanalysis II||(1979–2010)	Global (2.5° × 2.5°) (ca. 70 000 km^2^)¶¶	*T*_min_, *T*_max_, wind speed, precip, RH, wind speed, radiation	Lobell & Asner (2003), Nemani *et al*. (2003), Schlenker & Roberts (2009), Twine & Kucharik (2009)
			ERA-Interim Reanalysis (1989–2013)^**^	Global (1.5° × 1.5°) (ca. 25 000 km^2^)	*T*_min_, *T*_max_, wind speed, precip, RH, wind speed, radiation	Rötter (1993), de Wit *et al*. (2010)
	Interpolated from weather stations	Monthly	CRU05 (3.10)^††^, Univ. Delaware Climate Dataset (1961–2009)	Global (0.5° × 0.5°) (ca. 3000 km^2^)	*T*_min_, *T*_max_, total precip, no. of wet days, vapor pressure	Fischer *et al*. (2002), Foley *et al*. (2005), Bondeau *et al*. (2007), Lobell (2007), Lobell *et al*. (2008), Battisti & Naylor (2009), Licker *et al*. (2010), Lobell *et al*. (2011)
		Average 50-year monthly mean	WorldClim^‡‡^ (1950–2000)	Global(ca. 1 km^2^)	*T*_min_, *T*_max_, total precip, no. of wet days	Ortiz *et al*. (2008), Nelson *et al*. (2010)
	Satellite	Daily	NASA-Power^§§^(1983–2010) except precip (1997–2010)	Global 1° × 1° (ca. 12 000 km^2^)	*T*_min_, *T*_max_, precip, Tdew, radiation, RH	Lobell *et al*. (2010)

* Minimum temperature (*T*_min_), maximum temperature (*T*_max_), precipitation (precip), relative humidity (RH), incident solar radiation (radiation).

† High Plains Regional Climate Center (HPRCC). http://www.cma.gov.cn/english/.

‡ China Meteorological Administration (CMA). http://www.cma.gov.cn/english/.

§ German Weather Service (DWD. http://www.dwd.de/.

¶ National Oceanic and Atmospheric Administration (NOAA) Global Historical Climate Data-daily: http://www.ncdc.noaa.gov/oa/climate/ghcn-daily/.

|| National Center for Environmental Prediction/Department of Energy (NCEP). http://www.esrl.noaa.gov/psd/data/gridded/data.ncep.reanalysis2.html.

** ECMWF re-analysis (ERA). http://www.ecmwf.int/research/era/do/get/era-interim.

†† Climate Research Unit (CRU). http://badc.nerc.ac.uk/data/cru/.

‡‡ WorldClim. http://www.worldclim.org/.

§§ National Aeronautics and Space Administration (NASA). http://power.larc.nasa.gov/.

¶¶ Aproximate grid cell area near the equator.

Assessment of climate change impacts on future crop yield requires confidence in the simulated yields that are taken as a baseline. No previous studies, however, have compared how these baselines may vary depending on source of the global weather data used in the analysis. To fill this knowledge gap, we evaluated how well currently available global GWD perform when used as input for crop model estimates of Yp or Yw compared with similar simulations made with observed, high quality site-based weather data. Underpinning causes for observed differences in simulated yields were identified based on case studies in three major cropping systems: rainfed maize in US, irrigated rice in China, and rainfed wheat in Germany, which together are representative of 25% of global cereal grain supply (assuming German wheat production is representative of wheat production in northwest Europe). We also assessed capacity to simulate crop yields with publicly available weather station data that has greatest global coverage in terms of number and distribution of weather stations, which may provide another option for estimating current baselines and future crop yields in climate change studies.

## Materials and methods

### Databases selected for comparison

Weather data used as a benchmark for simulation of Yp or Yw were obtained from regional networks of meteorological stations that have complete daily records of weather data, and which also undergo rigorous quality control measures. Available data recorded by these weather stations, hereafter called ‘control weather data’ (CWD), include all daily time-step variables required to simulate Yp or Yw (see detailed description of the variables in the following section). CWD were taken from (i) the High Plains Regional Climate Center for rainfed maize in the USA (HPRCC, 2011); (ii) the China Meteorological Administration for irrigated rice in China (China Meterological Administration, 2009); and (iii) the German Weather Service for rainfed wheat in Germany (DWD, 2009). Four locations in each country were selected based on completeness of weather data records and location in regions with high density of crop production as identified by Van Wart *et al*. (2013a).

The GWDs selected for our study and one global weather station database are publically accessible, diverse in spatial and temporal resolution, and widely used in the published literature for estimating effects of climate change on food security (Table 1). The three GWDs include: (i) National Center for Environmental Prediction and Department of Energy's reanalysis II (NCEP/DOE) (Kanamitsu *et al.*, 2002); (ii) Climate Research Unit's high-resolution gridded dataset time series 3.1 (CRU) (New *et al.*, 2002); and (iii) National Aeronautics and Space Administration's POWER database (NASA), produced by the NASA Langley Research Center POWER Project funded through the NASA Earth Science Directorate Applied Science Program. A fourth database of location-specific weather data came from weather stations in the National Oceanic and Atmospheric Administration's Global Historical Climate Network-daily, hereafter called NOAA database (NCDC, 2011). In all cases the NOAA weather stations are distinct from the CWD stations although they are in close proximity. A description of spatial and temporal resolution of these weather databases, as well as their reported meteorological variables, is found in Table 1.

Gridded daily NCEP data are derived from a global climate model based on observed weather data from meteorological stations, ocean buoys, satellite data, and other sources (Kalnay *et al.*, 1996; Kanamitsu *et al.*, 2002). Gridded monthly CRU data are derived by interpolating weather data from 14 000 stations around the world using a thin-plate spline method which accounts for latitude, longitude, and elevation (New *et al.*, 2002; Mitchell and Jones, 2005). Gridded, daily NASA-POWER data are derived from satellite observations coupled with the Goddard Earth Observing System Model, an integrated system of models informed by observed data from multiple sources (satellite, ground stations, etc.).

Values calculated for each grid can serve as model input themselves or be understood as values located at the center of the grids. These grid-center values can be used to interpolate values at another location within the grid based on distances from that location to neighboring grid-centers. In the present study, separate simulations of Yp and Yw were performed for all crops based on gridded NCEP and CRU weather data using: (i) reported gridded data for the grid in which the meteorological weather stations were located; and (ii) data interpolated from center points of nearby grids to the location of the CWD meteorological stations by distance-based bilinear interpolation following the method described in Chang (2009).

**Fig 1:**
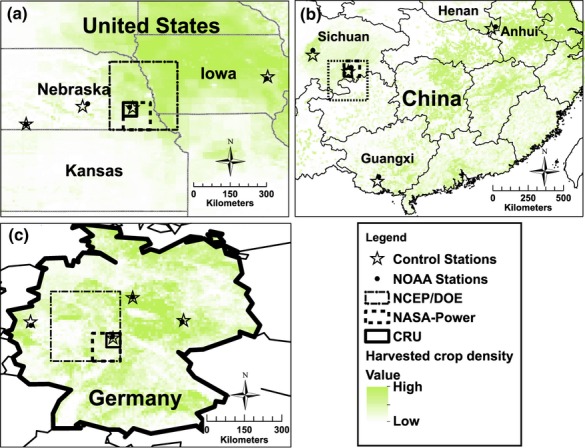
Locations of control weather stations, NOAA weather stations and size of NCEP/DOE, NASA-POWER, and Climate Research Unit (CRU) grids (shown for one of the control weather data sites) for (a) maize in the USA, (b) rice in China, and (c) wheat in Germany. Grid size is: 2.5° × 2.5° for NCEP, 1.0° × 1.0° for NASA, and 0.5° × 0.5° for CRU. Harvested crop area density is indicated by shaded areas on each map.

The NOAA database is an archive of daily historical weather observations from 40 000 meteorological stations around the world, the data of which have undergone several quality control measures (NCDC, 2011). Selected NOAA weather stations were located near CWD sites (Fig.1). Because the NOAA data do not contain values for daily solar radiation, which are critical for robust simulation of crop yields, NOAA data were coupled with satellite-derived NASA daily solar radiation (SR) to estimate Yp and Yw (hereafter called NOAA-SR). This approach was taken for two reasons. First, previous studies have found that simulation of crop yields using a combination of NASA-derived SR and weather station data for temperature and rainfall were in close agreement with simulations based on measured SR at the weather stations (White *et al.*, 2011b; Bai *et al.*, 2010). These studies demonstrate that NASA's SR, though gridded, is well correlated with SR observed at ground stations in topographically homogenous (i.e. flat) regions where field crops are typically grown. Second, use of NASA-derived SR to estimate Yp or Yw was in closer agreement with simulations based on measured SR compared with simulations based on SR estimated from temperature and/or sunshine hours (Van Wart *et al.*, 2013a).

### Yield simulations

Crop Yp and Yw were simulated using ORYZA2000 for rice (Bouman *et al.*, 2001), HybridMaize for maize (Yang *et al.*, 2004), and CERES-Wheat (Ritchie *et al.*, 1988), the latter embedded in dssat 4.0 (Jones *et al.*, 2003). Each of these crop simulation models have been well documented and validated against yields measured in field experiments that received optimal management (Ghaffari *et al.*, 2001; Bouman & van Laar, 2006; Grassini *et al.*, 2009). These models operate on a daily time-step; hence, they require daily weather data including incident SR and maximum and minimum temperature (*T*_max_ and *T*_min_, respectively) to simulate Yp. Simulation of Yw in rainfed cropping systems also requires precipitation and other variables needed to estimate reference evapotranspiration (ET_o_), including wind speed, dew point temperature, and/or relative humidity (RH). Simulated grain yields in this study are reported at standard moisture contents of 0.140, 0.155, and 0.135 kg H_2_O kg^−1^ grain for rice, maize and wheat, respectively, because this is comparable to yield records in global and national databases maintained by agencies such as USDA and FAO. Other input parameters necessary for simulating Yp or Yw for each crop at each location include soil properties (soil texture, soil depth, plant available soil water holding capacity), management practices (sowing date and plant population), and cultivar-specific (genotype) coefficients, which were taken from Van Wart *et al*. (2013a). These inputs were held constant for the simulation at each location regardless of the GWD data used as input to yield simulations (see Tables S1–S3).

Simulation of Yw for rainfed maize in USA required information on planting date, hybrid maturity, plant population, planting density, and soil properties (including soil texture and initial plant available soil water) as determined by Van Wart *et al*. (2013a). Maize planting dates were determined as average date from 2003–2008 for which the USDA's Risk Management Agency (RMA) reported 50% of maize area as planted for the counties in which CWD-sites were located (RMA, 2010). Seeding rate and hybrid maturity for the most commonly used hybrids were obtained from field researchers and seed company agronomists. The SSURGO database was used to identify the dominant agricultural soil within 100 km of control-sites based on area planted with maize as identified by the 2009 USDA crop data layer (USDA-NASS, 2009). Initial soil water at planting was assumed to be 100% field capacity, which is typical for most rainfed maize area in the US Corn Belt.

In China, multiple crops are planted in a single year on the same piece of land, as opposed to single cropping found in more temperate regions. In the present study, the dominant rice systems in the targeted locations were simulated, resulting in a total of six rice cropping systems by location combinations (see Tables S1–S3). Data used to simulate irrigated rice Yp using ORYZA2000 were provided by local agronomists in China, including sowing or transplanting date, hill spacing, and dominant rice cultivar for each cropping system as reported by Van Wart *et al*. (2013a). Soil data were not required because simulations assume irrigation is applied whenever the crop needs water regardless of soil type. Genotypic coefficients were determined for the dominant cultivar in each cropping system based on CWD and actual average transplanting, flowering and maturity dates reported by local agronomists. Calibration of genotypic coefficients was performed using drates software, which iteratively determines coefficients that give simulated estimates of date of rice flowering and maturity consistent with actual reported average rice flowering and maturity dates (Bouman *et al.*, 2001). Genotypic coefficients calibrated for CWD-sites were kept constant across the GWD-based simulations.

Simulation of rainfed winter wheat Yw required data on planting date, plant population, and soil properties. Average planting date and plant population at each site were obtained from the German Weather Service and local breeders and agronomists based on Van Wart *et al*. (2013a). Genotypic coefficients of the dominant wheat cultivars at each location were provided by Jans Bobert (Leibniz Centre for Agricultural Landscape Research). These genotypic coefficients were kept constant across simulations made with GWD and CWD data. Finally, soil water was assumed to be 100% field capacity at the start of the season (typical of rainfed wheat fields in Germany) and soil properties were retrieved from soil profile descriptions of dominant soil series reported by Hartwich *et al*. (1995).

Nineteen years of data were available for both the CWD and the four GWDs for all rice simulations and for two of the maize locations (1990–2008). The other two maize locations had CWD for 11 (1998–2008) and 14 years (1995–2008). The longest time-span of consecutive years available from NOAA stations in Germany at the time of this study was from 1983–1991. Because an unbiased analysis requires equivalent time-series for all weather databases, Yw simulations of wheat in Germany were performed over these nine consecutive years using CWD, NCEP, CRU, and NOAA-SR data. However, NASA-POWER data do not begin reporting rainfall until 1997, therefore, simulations of Yw of wheat in Germany and Yw in USA using NASA data were only performed for the years 1997–2008 and compared with CWD-based simulations for the same time interval. Total observations were *n* = 63 for rainfed maize in the USA (11–19 years, four sites), *n* = 76 for rice in China (19 years, four sites), and *n* = 36 for wheat in Germany (9 years, four sites).

### Quality control, temporal interpolation, and estimation of missing parameters

Crop models operate on a daily time-step, hence, daily weather data are required to simulate Yp or Yw. The NCEP, NASA, and CWD datasets contain daily values for the entire time-series included in this study. CRU monthly data require temporal interpolation, and raw NOAA station data require correction of missing or erroneous data. Cubic spline interpolation was used to derive daily *T*_min_, *T*_max_, vapor pressure, and percent cloud cover (a proxy for sunshine hours) from monthly CRU data. Daily rainfall data were generated from records of total monthly precipitation and monthly wet day records following the stochastic precipitation generation method described in Liu *et al*. (2009).

To achieve complete daily records for the NOAA data, it was necessary to identify and replace erroneous values and fill in missing values. A spatial regression test (SRT) was used to check weather data of each NOAA station based on whether each datum fell within a confidence interval calculated from a weighted regression estimate of each datum based on nearby station data (Hubbard *et al.*, 2007). For each of at least two stations closest to the station to be tested, a regression estimate is formed for each tested day and parameter (e.g. *T*_min_, *T*_max_, rainfall) based on the previous and preceding 15 days of data. A SRT estimated value for each datum is then calculated by weighting each regression estimate by the SE of the regression. If a tested station datum is missing or outside the confidence interval, calculated as the SRT estimate plus or minus 3 SDs (5 for precipitation), it is replaced by the SRT estimate. This method was found to outperform other quality-control methods over a wide variety of agro-climatic regions (Hubbard *et al.*, 2005; You *et al.*, 2008). Approximately 0.5% of all NOAA weather records required correction in the present study. In cases where a single daily record was missing from both the targeted and nearby stations, the average of the preceding and succeeding day was used to substitute the missing value (<0.01% of all weather records in the present study).

All GWDs required estimation of unreported parameters. Following Allen *et al*. (1998), wind speed was assumed to be equal to 2 m s^−1^ for CRU. RH was estimated using the Clausius-Clapeyron equation and SR was estimated using the Angstrom equation with percent cloud cover serving as a proxy for the ratio of actual sunshine duration to maximum possible sunshine duration (Foken & Nappo, 2008). Dew point temperature was estimated based on the Magnus-Tetens formula for both CRU and NCEP. ET_o_ was estimated based on Penman-Monteith-FAO equation for all databases.

### Evaluation of weather databases for simulation of Yp and Yw

Mean error (ME), root mean square error (RMSE), and coefficient of variation (CV) were calculated for simulated Yp or Yw based on each GWD and NOAA-SR as follows: 
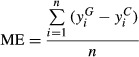
(1)

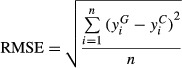
(2)

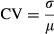
(3)

where 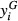 is the Yp or Yw simulated using data from a GWD for the *i*th site-year, 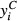 is Yp or Yw simulated using the CWD for the *i*th site-year, *n* is the number of site years, and *σ* and μ are standard deviation and mean, respectively, of simulated Yp or Yw. ME is a measure of average magnitude and bias (+ or −) of the error in simulated yield with GWDs or NOAA-SR compared with simulations with CWD. RMSE and %RMSE quantify average error on an absolute or relative basis compared to control mean values simulated with CWD respectively. CV is a measure of the relative variability in a distribution.

Forward stepwise regression was used to identify weather variables that best explain differences in Yp or Yw as simulated by the different weather data sources (Draper & Smith, 1981). The difference between simulated Yp or Yw based on CWD and a GWD or NOAA-SR was the dependent variable, while the difference between a given weather variable from the CWD and a GWD or NOAA-SR were independent variables. Four weather variables were examined: average daily *T*_min_ and *T*_max_, cumulative SR, and cumulative water deficit, defined here as precipitation minus ET_o_. Values of these weather variables were calculated for two crop phases: planting-to-anthesis and anthesis-to-physiological maturity for rice and wheat and planting-to-silking and silking-to-physiological maturity for maize. This resulted in a total of eight possible independent variables for inclusion in stepwise regression for each crop-country case for a given weather database. Only variables significant at *P*-value ≤0.05 were included in the final regression. Tests for co-linearity were null for all independent variables used in the regressions (*P* ≥ 0.05).

## Results

### Simulations with global weather databases

**Fig 2:**
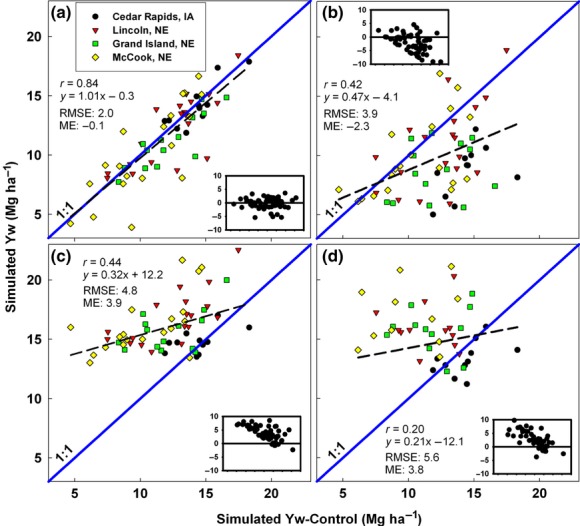
Simulated maize Yw across four sites in the USA Corn Belt using weather data from NOAA-SR (a), NCEP (b), Climate Research Unit (c), and NASA (d) plotted against simulated Yw based on a control weather database. Insets show deviations of points from the 1:1 line for each site and year for which yield was simulated with GWD or NOAA data. RMSE and mean error units are in Mg ha^−1^. Symbols represent different locations. NASA Yw simulations were performed for the time interval 1997–2007. Average water deficit (mm) over the maize growing season, as determined by simulations using control-data, was −42 (Cedar Rapids, IA), −135 (Lincoln, NE), −149 (Grand Island, NE), and −238 (McCook, NE).

**Fig 3:**
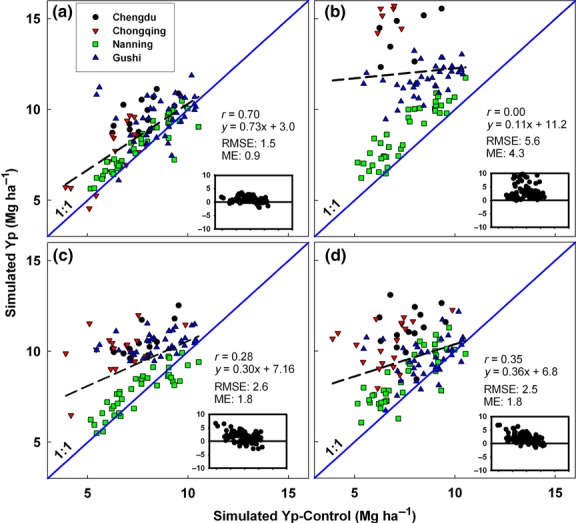
Simulated rice Yp across four sites in China using weather data from NOAA-SR (a), NCEP (b), Climate Research Unit (c), and NASA (d) plotted against simulated Yp based on a control weather database. Insets show deviations of points from the 1:1 line for each site and year for which yield was simulated with GWD or NOAA data. RMSE and mean error units are in Mg ha^−1^. Symbols represent different locations and cropping systems within each location. Site elevation (m) is 506 (Chengdu), 305 (Chongqing), 38 (Gushi), and 124 (Nanning).

**Fig 4:**
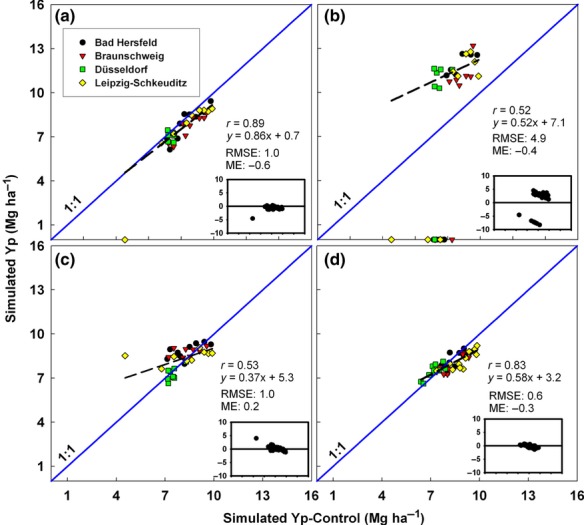
Simulated wheat Yw across four sites in Germany using weather data from NOAA-SR (a), NCEP (b), Climate Research Unit (c), and NASA (d) plotted against simulated Yw based on a control weather database. Insets show deviations of points from the 1:1 line for each site and year for which yield was simulated with GWD or NOAA data. RMSE and mean error (ME) units are in Mg ha^−1^. NASA Yw simulations were performed from 1997–2007. Symbols represent different locations. Note that site-years affected by frost have points on the *x*-axis at 0 Mg ha^−1^ and these Yw values were taken into account in all statistical calculations of RMSE and ME.

On average, simulated yields were overestimated by more than 1.5 t ha^−1^ in six of nine cases when based on data from gridded GWD compared with the simulated yields using CWD (Figs 2–4). Of particular note was the average upward bias of about 4.0 t ha^−1^ for Yw of US maize estimated by CRU and NASA, and for Yp of rice in China by NCEP. However, the bias between gridded GWD and CWD based simulations was not consistent. For example, except for NASA based simulations in Germany, simulations in China and Germany made with GWD data overestimated Yp or Yw. But NCEP-based simulations of Yw for maize in the USA had a negative bias of more than 1.0 t ha^−1^. While CRU based rainfed maize Yw simulations tended to overestimate Yp and Yw at high yield levels, NCEP and NASA tended to overestimate Yp at lower yield levels. In contrast, simulated yields using NOAA-SR weather data were in reasonably close agreement with yields simulated with CWD, although irrigated rice Yp in China had a modest overestimation of Yw (ME = 0.9 Mg ha^−1^). On average, %RMSE for Yp and Yw simulations based on gridded GWD was 45% and 33%, respectively, compared with 19% and 14% for NOAA-SR-based simulations. Likewise, the degree of correlation between Yp or Yw estimated by NOAA-derived weather data and the CWD was quite high in all cases, ranging from Pearson correlation r values of 0.70 for rice in China to 0.89 for wheat in Germany. Correlations with CWD values for Yp or Yw based on GWD were much poorer and sometimes not statistically significant.

Yield simulations using gridded GWDs from Chongqing, Chengdu, and Gushi were not well-correlated with simulations based on CWD. Two of these locations (Chongqing and Chengdu) are located in regions with heterogeneous landscapes in which rice is grown in large river valleys surrounded by mountains. Such heterogeneity further exacerbated the magnitude of error in estimates of Yp based on gridded GWD (Fig.3; Table S4). Hence, gridded weather data, assuming uniform distribution of weather variables over the entire grid, are clearly disadvantaged when used to predict crop yields in such heterogeneous grids—especially in GWD with large grid size like NCEP.

Compared with simulations using weather data based at grid centers, interpolation from grid centers to station locations had a negligible effect on accuracy of all CRU-based simulations for all sites (difference in RMSE <3%). The effect was similarly negligible for rainfed maize and rainfed wheat simulations based on NCEP data. However, for NCEP simulations of Yp of rice in China, interpolation of data dramatically increased the bias. Simulations of rice Yp using interpolated NCEP data had an RMSE which was 47% larger than rice Yp simulations made using noninterpolated NCEP data. We speculate this large differences between simulations made using interpolated and noninterpolated NCEP weather data are due to the large size of NCEP's 70 000 km^2^ grids and the heterogeneous landscape in some of the grids included in this study (see Table S3).

### Reasons for bias in simulated yields with global weather databases

**Fig 5:**
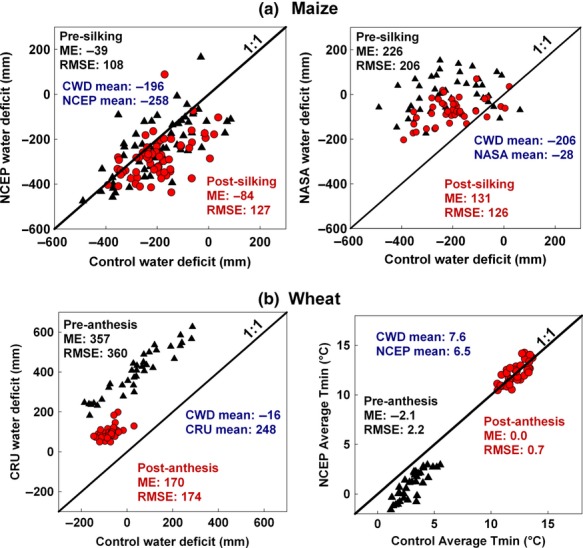
Four panel figure comparing reported weather data from control and GWDs during pre- (black triangles) and post-silking (red circles) for maize (a), and pre- and post-anthesis in wheat (b).

Stepwise multiple regression helped assess the causes of underlying bias in estimates of Yp and Yw using simulations with GWD, especially for rainfed maize and wheat, which are grown in regions with relatively uniform topography in the USA and Germany respectively (see Table S5 for a summary of GWD and NOAA-SR weather data biases). The range in average annual precipitation, however, differs markedly among CWD sites in Germany (500 and 850 mm) the USA (450–900 mm) and rainfall does not replace evapotranspiration in much of the western Corn Belt where the CWD sites are located (Grassini *et al.*, 2009). As a result, estimated water deficit and solar radiation had a large influence on discrepancies between Yw estimated by GWDs and CWD (Table 2). In general, the sign of coefficients in Table 2 are indicative of the relationship between that variable and yield. For instance, a positive sign for water deficit indicates that as this variable increases (less precipitation and more ET_o_) so do the deviations in simulated yields with a GWD as compared with simulations using the CWD. The closer GWD and NOAA-SR based simulated yields were to CWD based simulations (i.e. low RMSE and ME as shown in Figs 2–4), the poorer the explanatory power of the final regression model. For example, the water deficit calculated over the USA simulated maize growing season was 76% and 86% smaller for simulations based on CRU (data not shown) and NASA data, respectively, compared with those based on CWD (Fig.5a). Similarly, water deficit was 31% larger with NCEP maize simulations than with CWD, especially during the post-silking phase in which water deficit was 43% larger than the CWD (see Figs S1–S12).

**Table 2:** Summary of stepwise multiple regression of difference between Yp or Yw simulated using control and global weather databases regressed on the difference between each of control and global weather database values for average daily *T*_max_, average daily *T*_min_, cumulative solar radiation and cumulative water deficit during pre- and post-anthesis (pre-A and Post-A) in wheat and rice and pre- and post-silking in maize (Pre-S and Post-S). Results include significance of variables, regression coefficients of the variables, percent of total variation explained by each independent variable (explanatory power, % of total Type I sum of squares), and the adjusted *R*^2^ (Adj. *R*^2^) and *F*-test statistic for the stepwise regression

Database	Independent variables†	Coefficient‡	Explanatory power (%)	Adjusted *R*^2^	*F*-test
Maize					
NOAA	Post-S solar radiation*	0.005	11		
	Post-S water deficit***	0.008	16	0.25	11.1***
NCEP	Post-S solar radiation***	0.011	29		
	Post-S water deficit***	0.024	49	0.77	105.5***
CRU	Post-S average daily *T*_max_***	−1.412	33		
	Pre-S water deficit**	−0.005	17		
	Post-S water deficit**	0.015	13	0.61	33.5***
NASA	Post-S solar radiation***	0.011	64		
	Post-S water deficit***	0.030	22	0.85	136.2***
Rice					
NOAA	Post-A solar radiation***	0.005	12	0.11	14.1***
NCEP	Pre-A average daily *T*_max_***	−0.879	45		
	Post-A solar radiation***	−0.002	14	0.58	76.6***
CRU	Post-A average daily *T*_max_*	−0.379	5	0.04	5.5*
NASA	Post-A average daily *T*_max_**	−0.135	24		
	Pre-A solar radiation***	0.005	10	0.33	27.6***
Wheat					
NOAA	Pre-A solar radiation***	0.006	36	0.34	19.1***
NCEP	Pre-A average daily *T*_min_***	3.921	38	0.36	21.0***
CRU	Pre-A average daily *T*_min_***	−0.876	30		
	Post-A solar radiation*	0.005	8	0.34	10.2***
NASA	Pre-A solar radiation***	0.004	44	0.43	32.9***

† Variables were significant at

* *P* < 0.05

** *P *<* *0.01, and

*** *P* < 0.001.

‡ Coefficients reported are b values from the multiple regression equation: *y *= *a *+ *b*_1_*x*_1_ + *b*_2_*x*_2_ + *b*_3_*x*_3_ + … + *e*.

In some cases, differences between a specific weather variable in GWD and CWD did not have a large impact on estimates of yield potential because the variable in question was not a sensitive parameter. For example, although the water deficit was grossly overestimated for wheat in Germany in the CRU database, this bias did not have a large influence on the discrepancy in Yw estimates because rainfall is generally adequate for rainfed wheat in most of Germany (Figs 4 and 5b). Given adequate rainfall, the differences in simulated wheat yields were attributed more to differences in temperature and solar radiation (Table 2). For example, average pre-anthesis daily minimum temperature was lower in NCEP data compared with CWD data (0.9 °C vs. 3.0 °C). In some cases these low temperatures induced simulated frost-kill while in others they increased the pre-anthesis growth period and allowed for greater dry-matter accumulation by the time of anthesis, which increased grain set and final yield.

## Discussion

The twelve sites evaluated in this study included simulations of the three most important cereal crop species, in three major crop producing countries with very different climates and water regimes. Assessment of different sources of weather data for their capacity to simulate crop yields across this diversity of crops and environments gives confidence that findings from this study can be generalized to other major crop production regions. Results presented here document that GWDs, such as NCEP, CRU, or NASA, do a poor job of simulating Yp and Yw of rice, wheat, and maize. In contrast, simulations of crop yields based on NOAA-SR data, derived from actual weather stations, outperformed simulations made using the GWDs in nearly all cases especially for topographically diverse regions or where water deficit is a major limiting factor to rainfed crop production. Reasons for discrepancy between simulated Yp or Yw using GWDs vs. simulations using location-specific, high-quality weather data were attributed to biases in temperature, SR, and/or degree of water deficit in the GWDs.

Climate heterogeneity in GWDs is smoothed by interpolations or modeling, which may not appropriately capture topographic features affecting climate (Daly, 2006). Temporal interpolations, such as deriving daily data from monthly average values (e.g. CRU) are also problematic as they likewise attenuate the degree of weather event variegation, especially for extreme events. Using interpolation from grid-centers of a GWD to actual location of the control weather stations in the CWD did little to remove these biases or to improve the accuracy of yield simulation. Use of gridded GWD data can therefore lead to erroneous conclusions about the impact of climate change. It may be argued that differences in weather data do not have a large impact on long-term average crop yield estimates based on simulation if differences are random and cancel each other out over time or in cases where crop performance is not sensitive to a specific weather parameter (such as rainfall in Germany). However, food security and vulnerability of future populations will depend on annual variability of global crop yields as well as long-term average yields (Schmidhuber & Tubiello, 2007). Furthermore, inability to reproduce interactions between environment and management under current weather raises the question of whether these databases or those derived from them should be used in studies aiming at reproducing the impact of future weather on management adaptations to climate change.

The GWDs compared in this study are being used to derive climate change scenarios, such as those found in the IPCC 4th assessment, which in turn are used in analysis of the impact of future climate change on crop yields (IPCC, 2007; Battisti & Naylor, 2009). These GWDs do not provide a reliable or realistic baseline of crop yield simulations nor will climate change scenarios based on data from these GWDs. Such climate change scenarios do not produce credible representations of location-specific climate nor even climate at larger scales (Masson & Knutti, 2011; Ramirez-Villegas & Challinor, 2012). In this article, we extend these results to evaluate capacity of these GWDs to simulate crop yields.

Credible assessment of the impact of future climate on food production depends on ability to estimate crop yields accurately under a wide array of climates, cropping systems, and water regimes. The poor performance of GWDs in estimating crop yields as shown in this study calls into question the many prior evaluations of climate change impact on crop production based on use of GWDs (Table 1) (see also White *et al.*, 2011a). Because land use change is closely linked to agriculture, accurate estimates of crop yield levels have a large impact on future land-use and emissions from the agricultural production sector (Balmford *et al.*, 2005). Hence, there are trade-offs between spatial granularity and accuracy of crop management and weather data and need for complete global terrestrial coverage (Bondeau *et al.*, 2007; Ciais *et al.*, 2011).

Given results of this manuscript, estimates of crop production should be based on actual data from ground weather stations that report the key weather variables that drive crop growth and yield, including daily maximum/minimum temperature, rainfall, and SR. For ground weather stations that do not report SR (such as the NOAA station network), SR from NASA can be used in combination with the reported daily temperature and rainfall. If location-specific, daily weather data are not available, and assuming relatively flat topography typical of many major crop-producing regions, nearby data within 50–100 km would presumably be more appropriate for use in crop models than grid-based data. Use of point-based weather station data to estimate regional and global impact of climate change on food production capacity is challenged, however, by the need to upscale results. Use of agro-climatic zones provides a means to perform this aggregation for upscaling although the required degree of geospatial granularity remains an issue still to be addressed (Wood & Pardey, 1998; Van Wart *et al.*, 2013b).

Also at issue is how to achieve complete terrestrial coverage in global assessments of climate change impact on future food security, which includes regions not currently inhabited or producing crops. Availability of weather data from such regions is sparse at best and often lacking entirely. Use of GWD is the only current option. We therefore propose that global analyses using GWD should be complemented with studies based on upscaling from point-based weather station data for the major centers of current crop production.

## Supporting Information

Figure S1 Comparison of weather data from control and NOAA- solar radiation during pre- (black triangles) and post- (red circles) silking of simulated rainfed maize in USA.

Figure S2 Comparison of weather data from control and NCEP global weather database during pre- (black triangles) and post- (red circles) silking of simulated rainfed maize in USA.

Figure S3 Comparison of weather data from control and Climate Research Unit global weather database during pre- (black triangles) and post- (red circles) silking of simulated rainfed maize in USA.

Figure S4 Comparison of weather data from control and NASA global weather database during pre- (black triangles) and post- (red circles) silking of simulated rainfed maize in USA.

Figure S5 Comparison of weather data from control and NOAA- solar radiation during pre- (black triangles) and post- (red circles) anthesis of simulated irrigated rice in China.

Figure S6 Comparison of weather data from control and NCEP global weather database during pre- (black triangles) and post- (red circles) anthesis of simulated irrigated rice in China.

Figure S7 Comparison of weather data from control and Climate Research Unit global weather database during pre- (black triangles) and post- (red circles) anthesis of simulated irrigated rice in China.

Figure S8 Comparison of weather data from control and NASA global weather database during pre- (black triangles) and post- (red circles) anthesis of simulated irrigated rice in China.

Figure S9 Comparison of weather data from control and NOAA- solar radiation during pre- (black triangles) and post- (red circles) anthesis of simulated rainfed wheat in Germany.

Figure S10 Comparison of weather data from control and NCEP global weather database during pre- (black triangles) and post- (red circles) anthesis of simulated rainfed wheat in Germany.

Figure S11 Comparison of weather data from control and Climate Research Unit global weather database during pre- (black triangles) and post- (red circles) anthesis of simulated rainfed wheat in Germany.

Figure S12 Comparison of weather data from control and NASA global weather database during pre- (black triangles) and post- (red circles) anthesis of simulated rainfed wheat in Germany.

Table S1–S3 Management parameters used in simulation models at four sites in three countries for three crops. Dates of planting, transplanting, and physiological maturity are reported as day of the year (DOY). Maize crop variety expressed in relative maturity days (CRM). **Table S4.** Elevation within 100 km of simulation sites in China (m). Source: CGIAR-CSI (2006): NASA Shuttle Radar Topographic Mission available for download at: http://srtm.csi.cgiar.org/ **Table S5.** Mean error (ME) and root mean square error (RMSE) using different global weather databases compared with local, high-quality control data during the growing season time period used in simulations of crop yields at each of four sites for rainfed maize in USA, irrigated rice in China, and rainfed wheat in Germany.
