# Supplementary figures and images for: Impact of derived global weather data on simulated crop yields
Source: Glob Chang Biol. 2013 Sep 24;19(12):3822–34. doi: 10.1111/gcb.12302 (PMC4288967; doi:10.1111/gcb.12302)

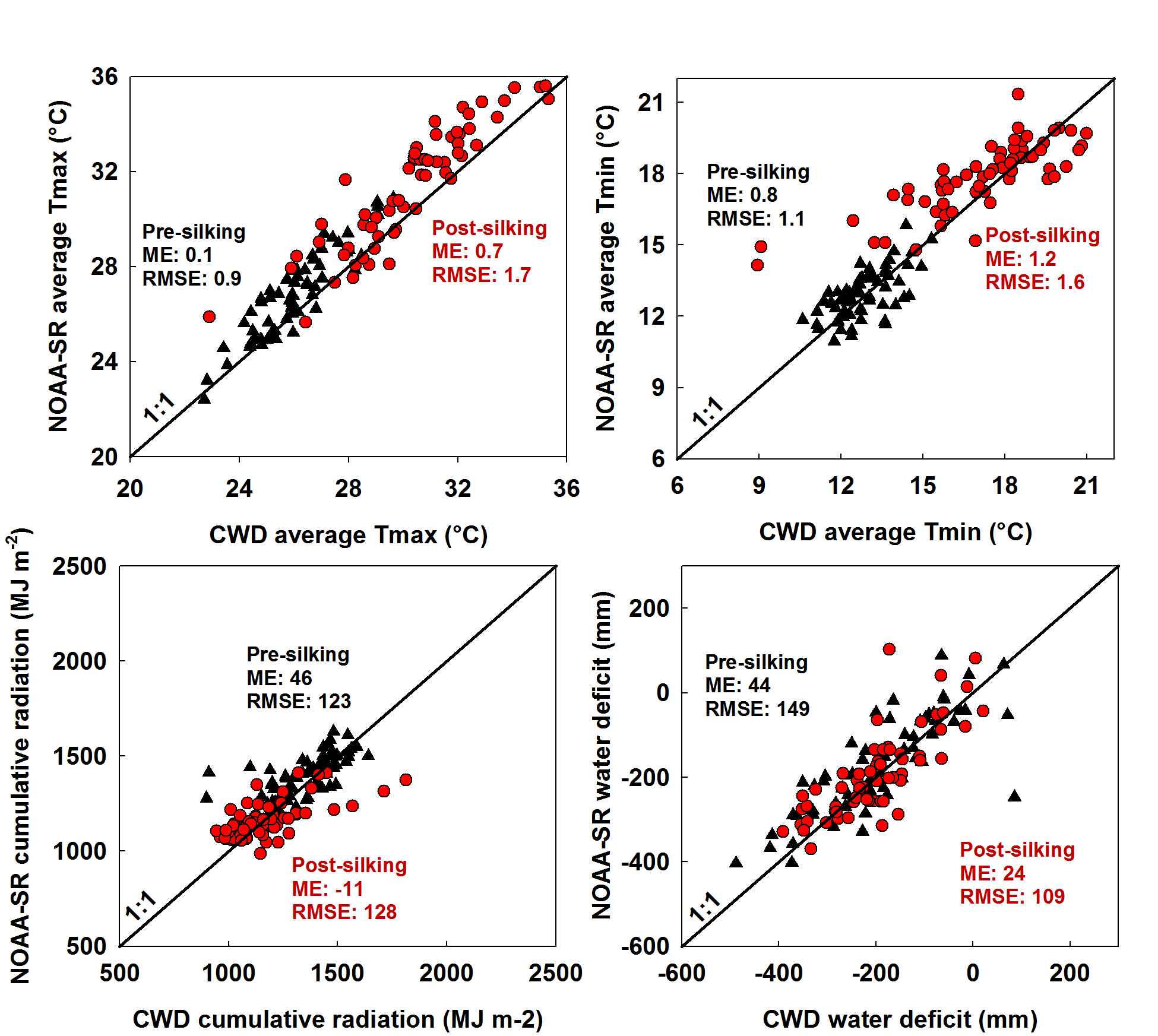

Supplement: Figure S1 — Comparison of weather data from control and NOAA- solar radiation during pre- (black triangles) and post- (red circles) silking of simulated rainfed maize in USA. [file gcb0019-3822-SD1.tif]

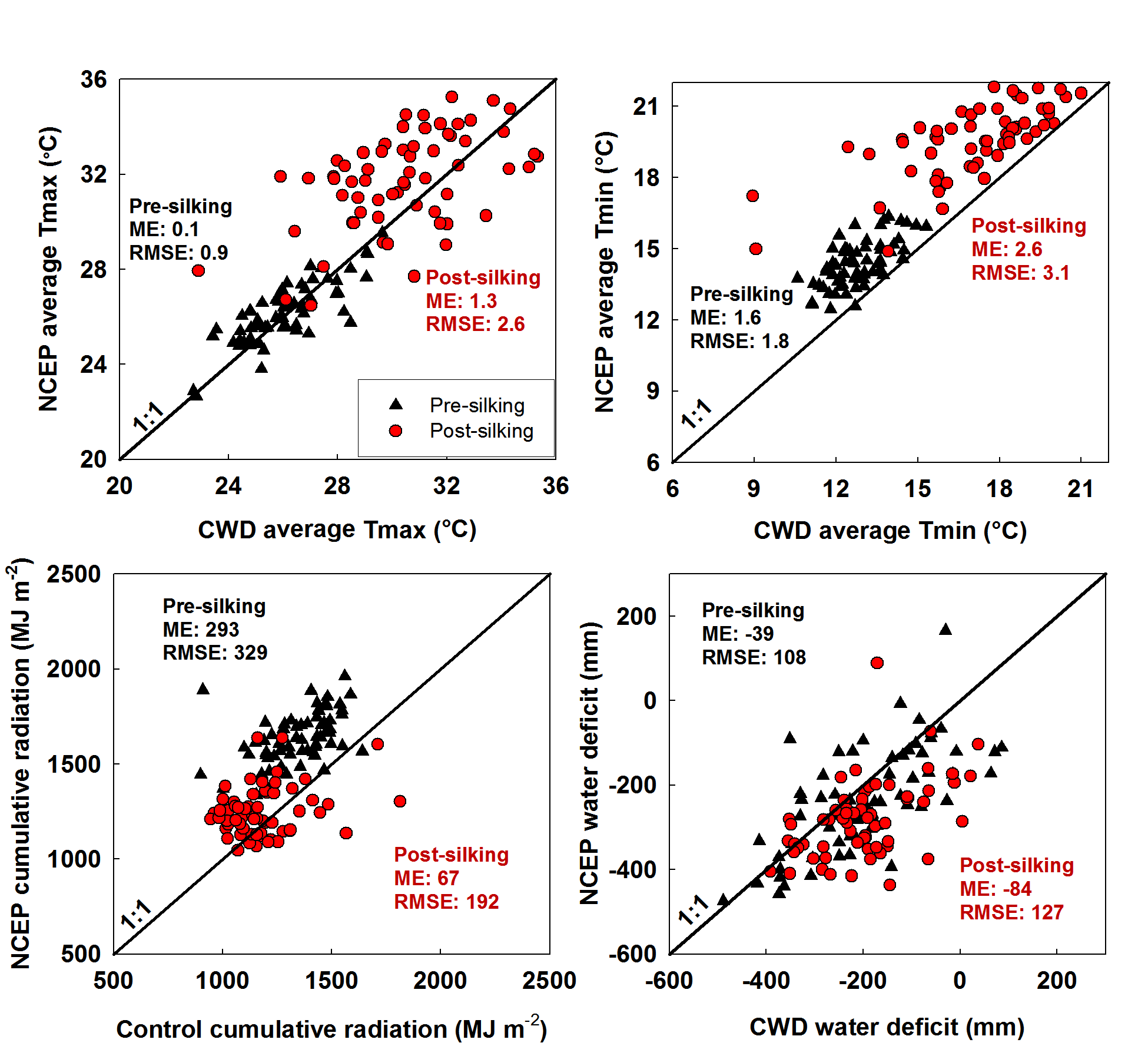

Supplement: Figure S2 — Comparison of weather data from control and NCEP global weather database during pre- (black triangles) and post- (red circles) silking of simulated rainfed maize in USA. [file gcb0019-3822-SD2.tif]

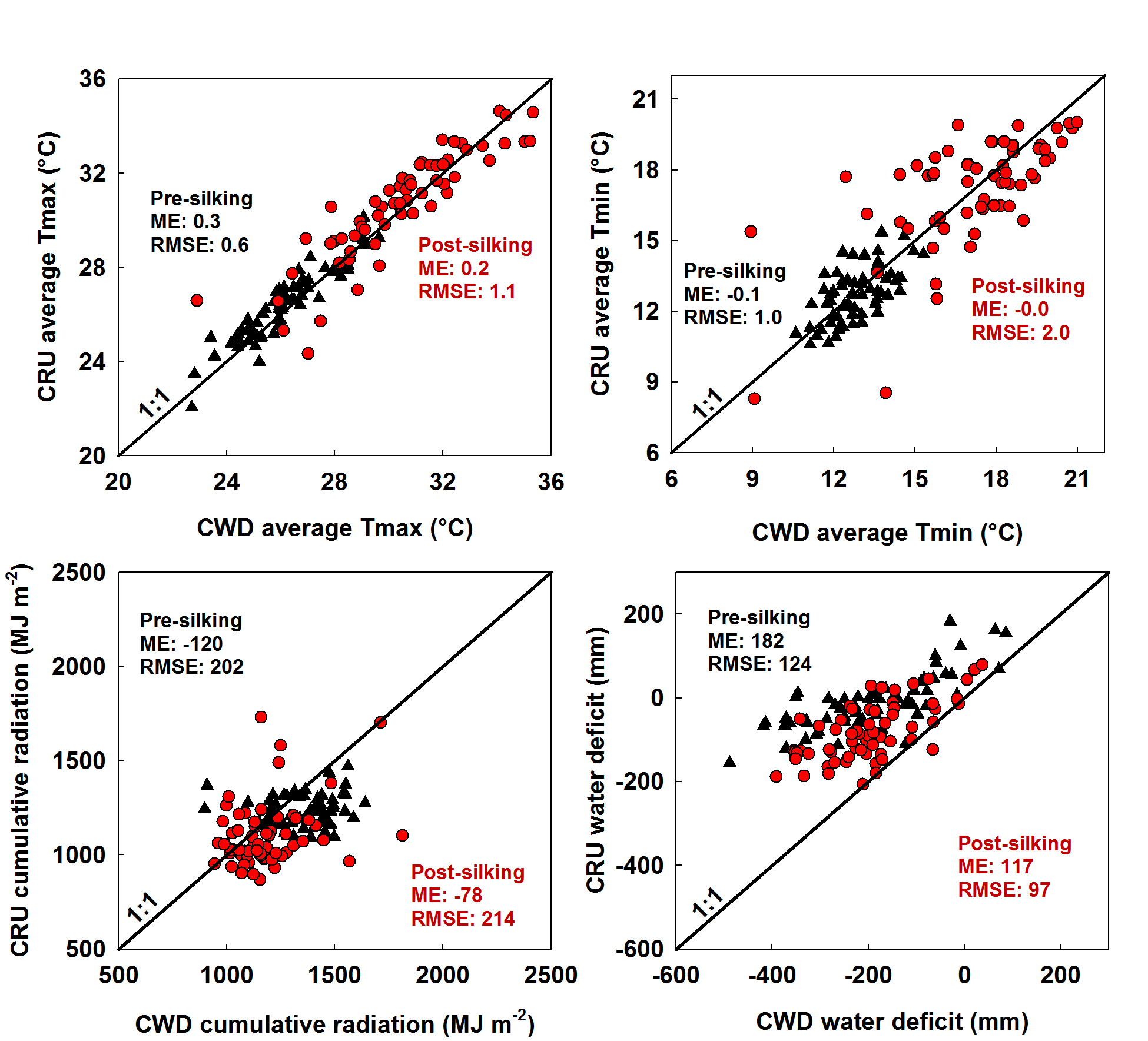

Supplement: Figure S3 — Comparison of weather data from control and Climate Research Unit global weather database during pre- (black triangles) and post- (red circles) silking of simulated rainfed maize in USA. [file gcb0019-3822-SD3.tif]

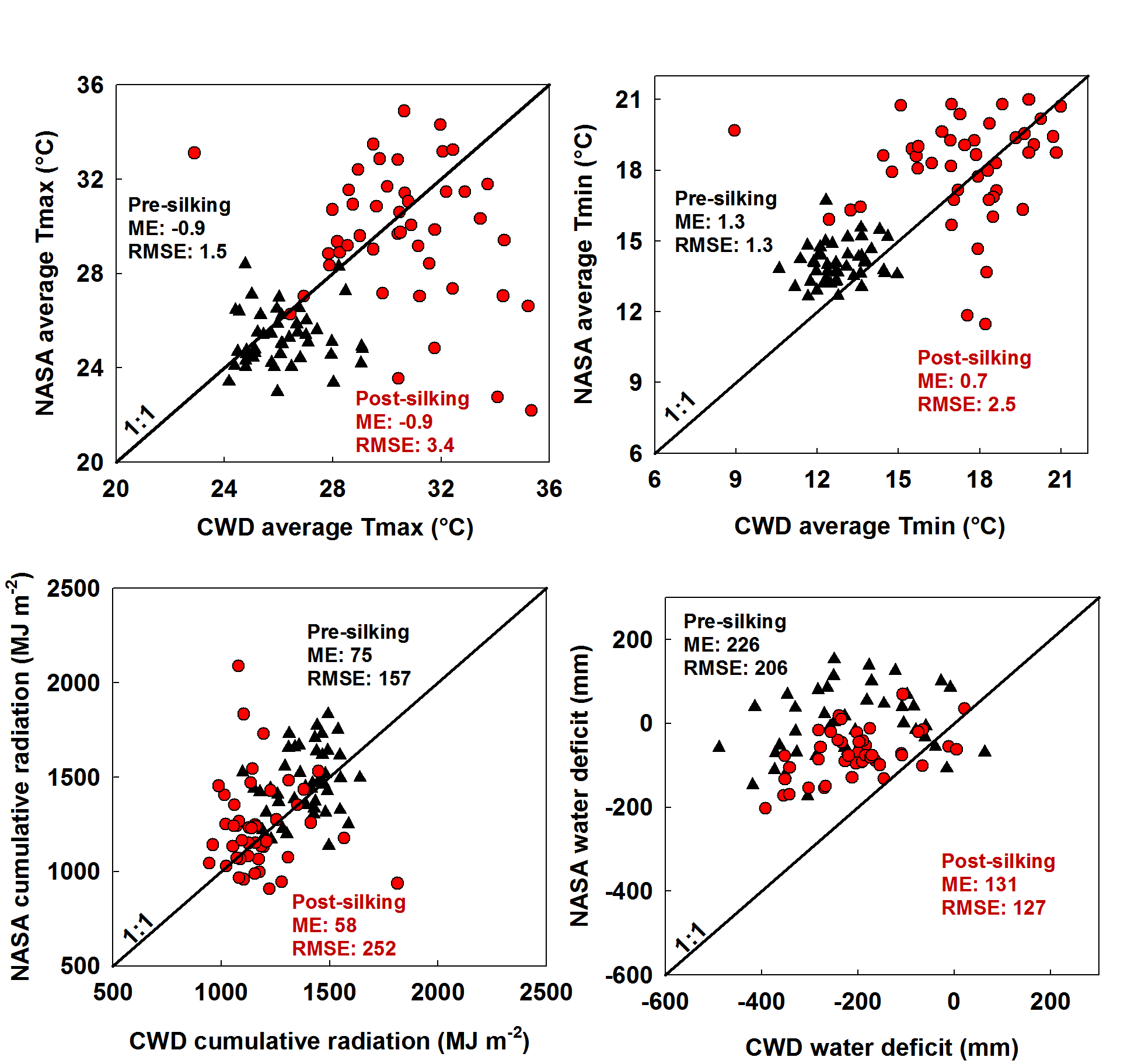

Supplement: Figure S4 — Comparison of weather data from control and NASA global weather database during pre- (black triangles) and post- (red circles) silking of simulated rainfed maize in USA. [file gcb0019-3822-SD4.tif]

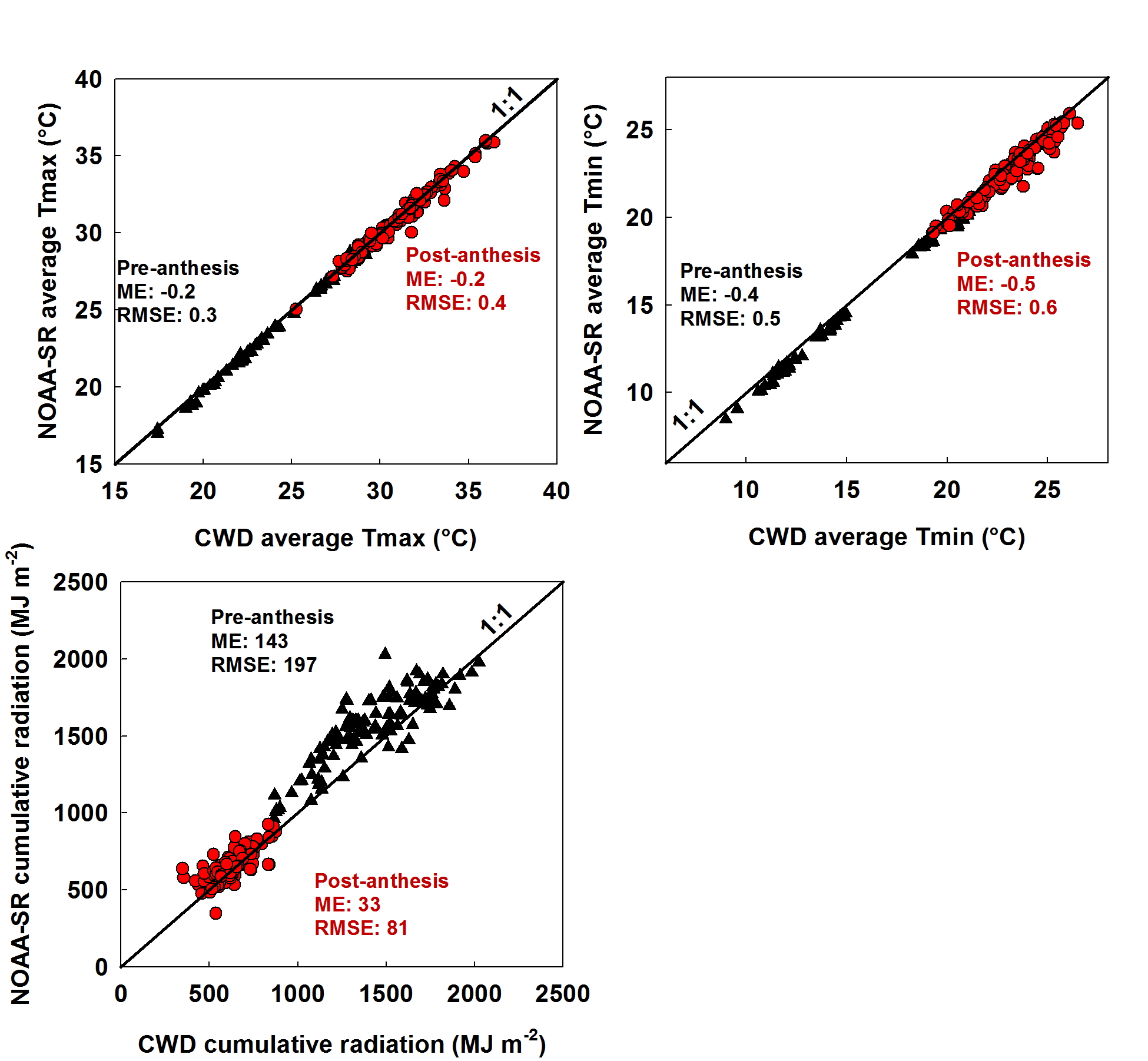

Supplement: Figure S5 — Comparison of weather data from control and NOAA- solar radiation during pre- (black triangles) and post- (red circles) anthesis of simulated irrigated rice in China. [file gcb0019-3822-SD5.tif]

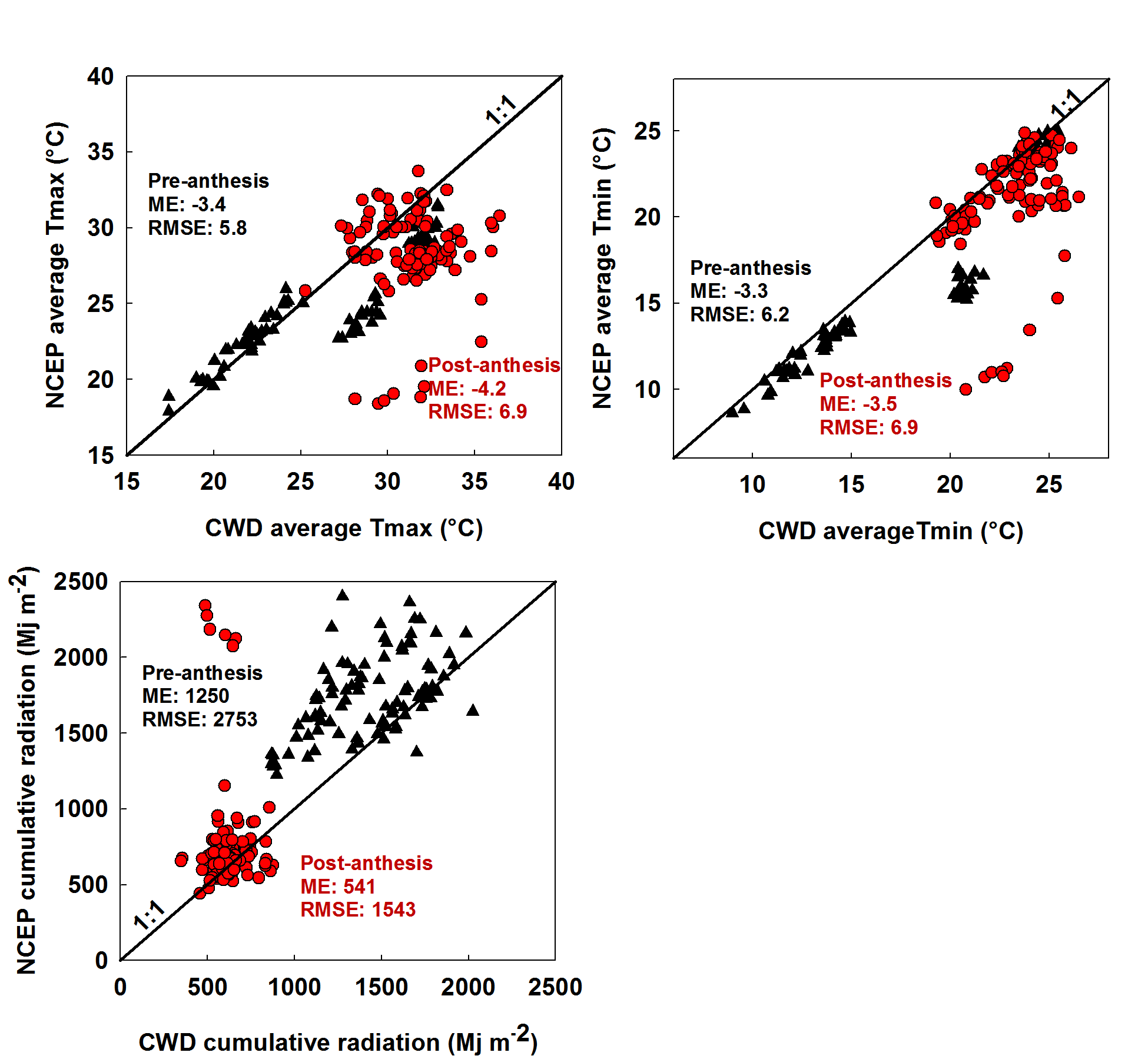

Supplement: Figure S6 — Comparison of weather data from control and NCEP global weather database during pre- (black triangles) and post- (red circles) anthesis of simulated irrigated rice in China. [file gcb0019-3822-SD6.tif]

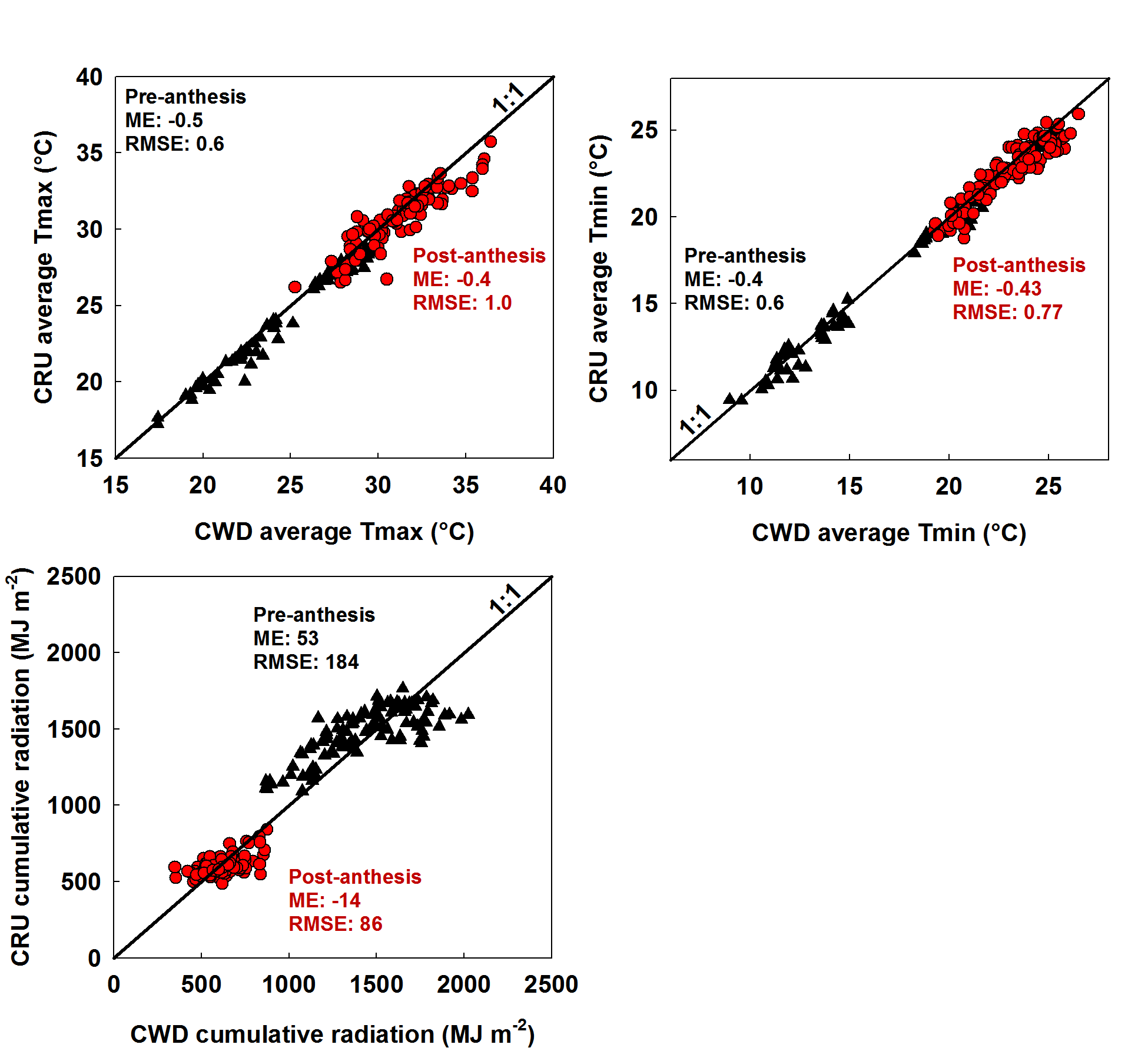

Supplement: Figure S7 — Comparison of weather data from control and Climate Research Unit global weather database during pre- (black triangles) and post- (red circles) anthesis of simulated irrigated rice in China. [file gcb0019-3822-SD7.tif]

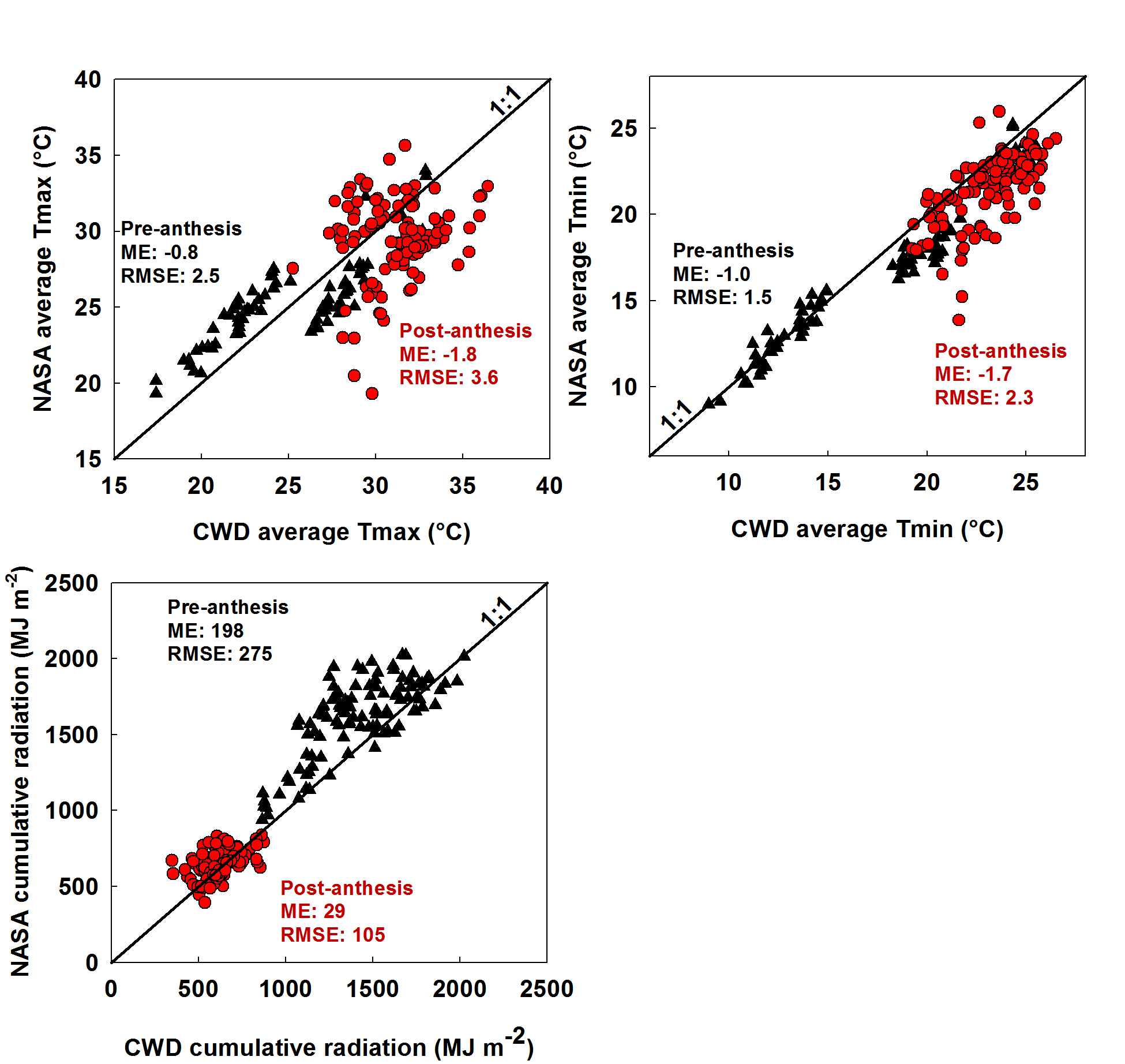

Supplement: Figure S8 — Comparison of weather data from control and NASA global weather database during pre- (black triangles) and post- (red circles) anthesis of simulated irrigated rice in China. [file gcb0019-3822-SD8.tif]

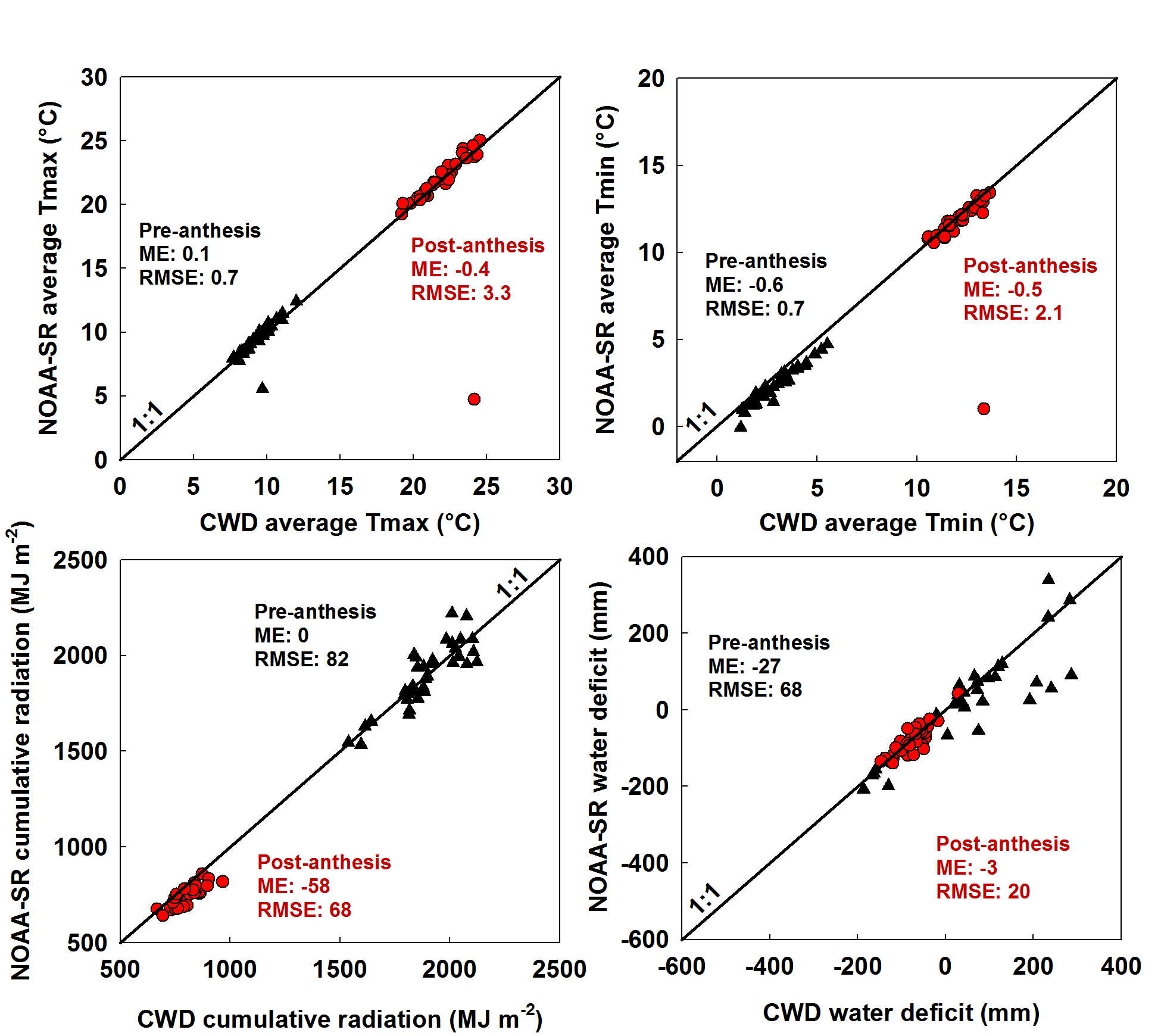

Supplement: Figure S9 — Comparison of weather data from control and NOAA- solar radiation during pre- (black triangles) and post- (red circles) anthesis of simulated rainfed wheat in Germany. [file gcb0019-3822-SD9.tif]

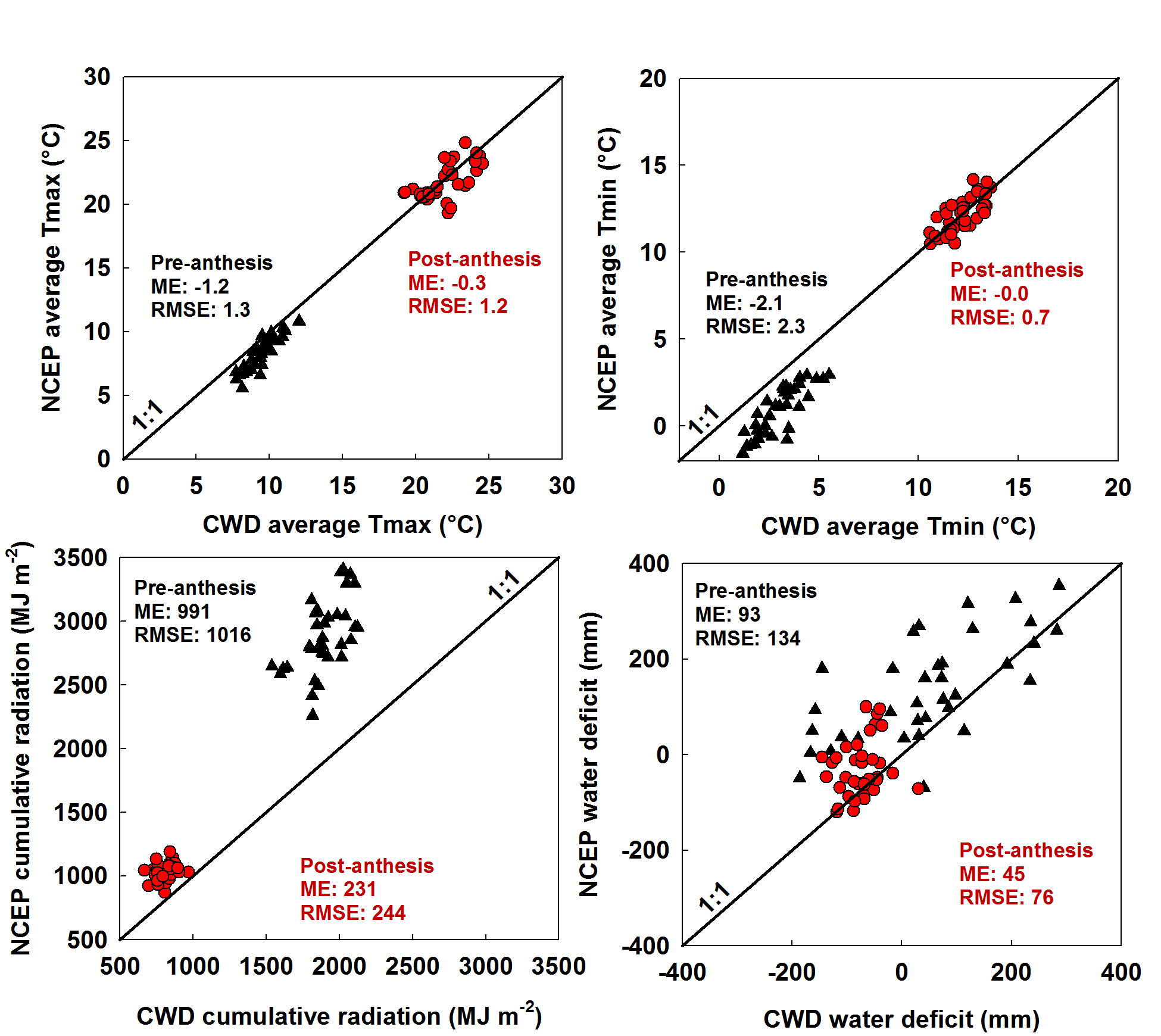

Supplement: Figure S10 — Comparison of weather data from control and NCEP global weather database during pre- (black triangles) and post- (red circles) anthesis of simulated rainfed wheat in Germany. [file gcb0019-3822-SD10.tif]

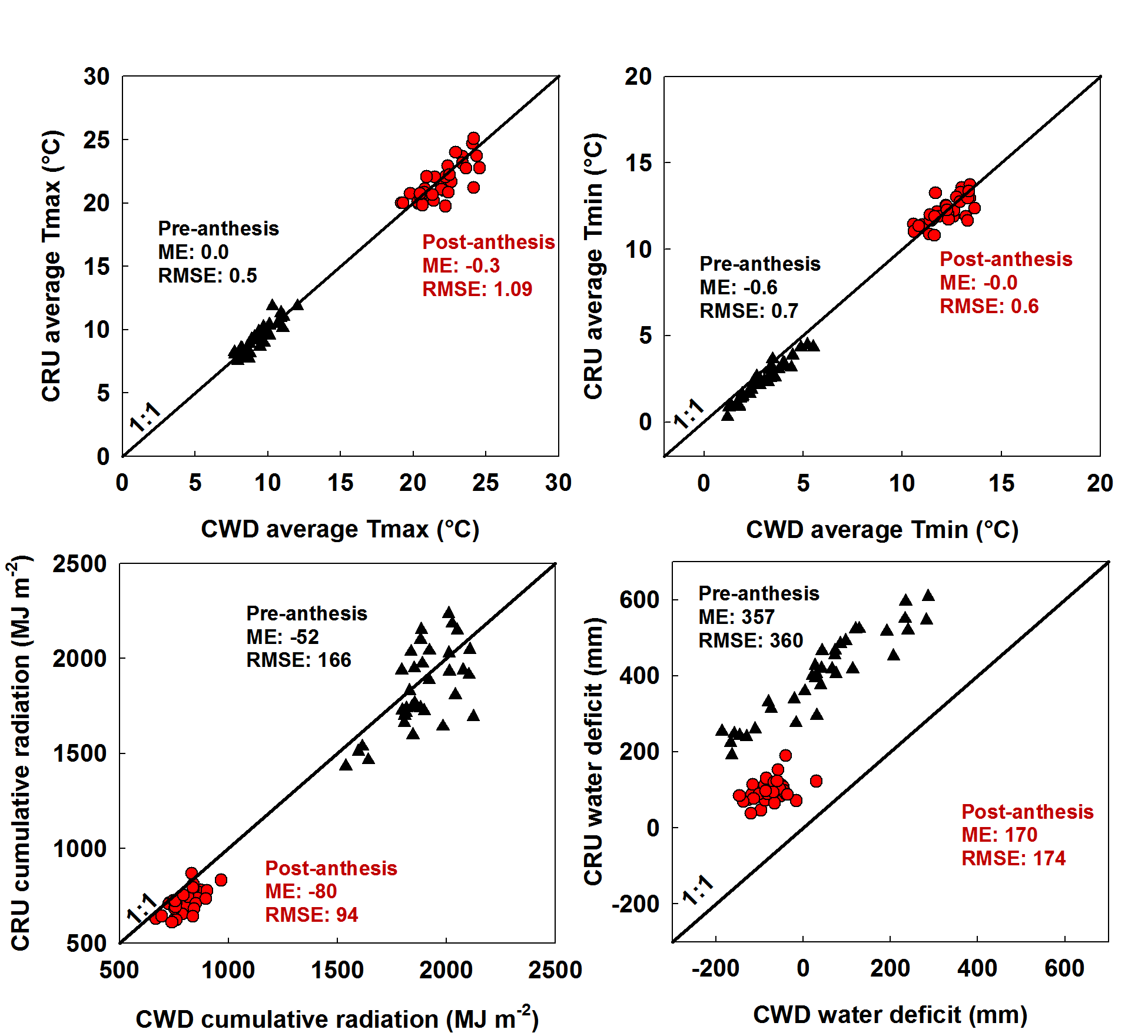

Supplement: Figure S11 — Comparison of weather data from control and Climate Research Unit global weather database during pre- (black triangles) and post- (red circles) anthesis of simulated rainfed wheat in Germany. [file gcb0019-3822-SD11.tif]

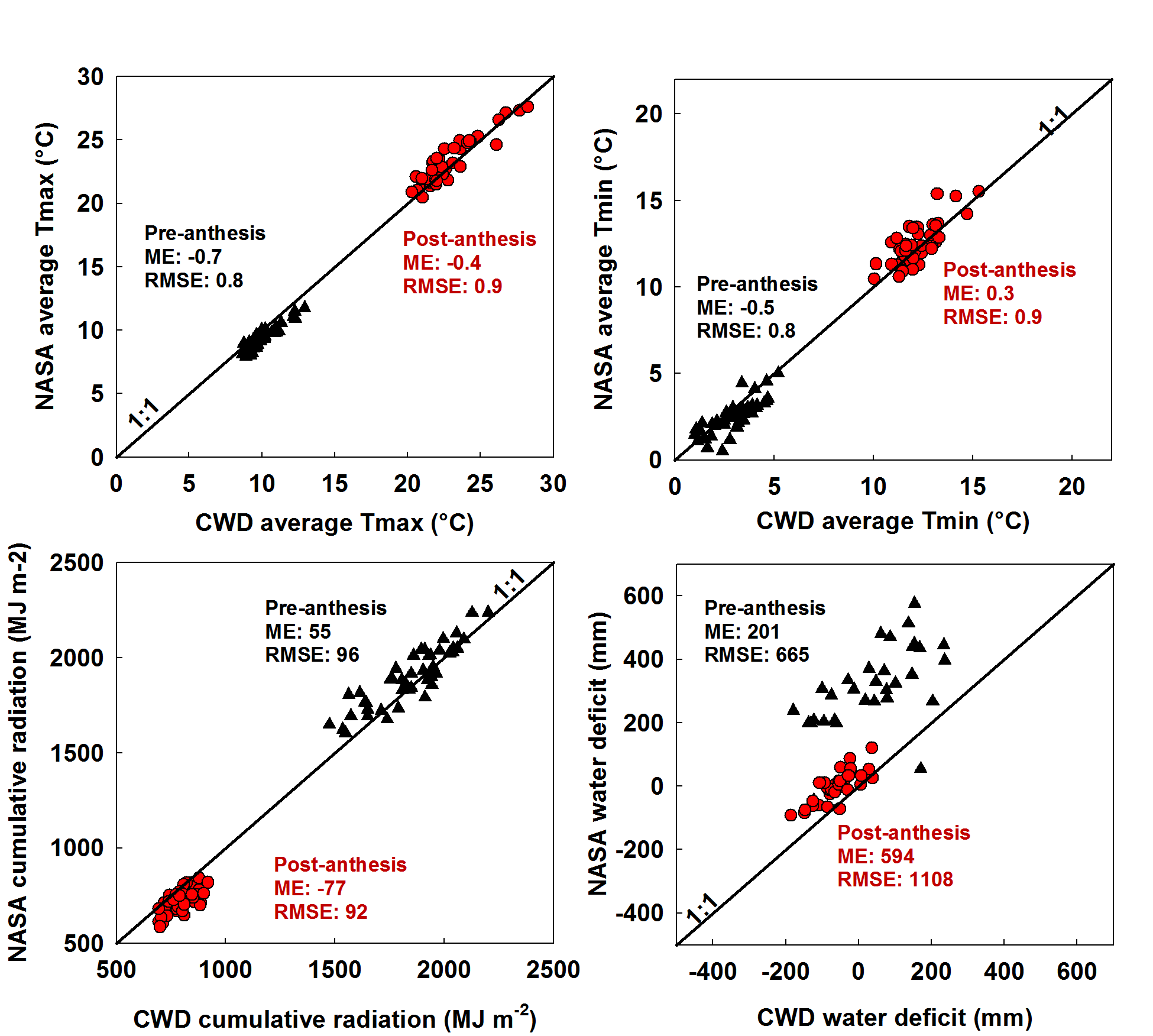

Supplement: Figure S12 — Comparison of weather data from control and NASA global weather database during pre- (black triangles) and post- (red circles) anthesis of simulated rainfed wheat in Germany. [file gcb0019-3822-SD12.tif]
