# Supplementary material for: Impact of derived global weather data on simulated crop yields
Source: Glob Chang Biol. 2013 Sep 24;19(12):3822–34. doi: 10.1111/gcb.12302 (PMC4288967; doi:10.1111/gcb.12302)
Supplement: Table S1–S3 — Management parameters used in simulation models at four sites in three countries for three crops. Dates of planting, transplanting, and physiological maturity are reported as day of the year (DOY). Maize crop variety expressed in relative maturity days (CRM). Table S4. Elevation within 100 km of simulation sites in China (m). Source: CGIAR-CSI (2006): NASA Shuttle Radar Topographic Mission available for download at: http://srtm.csi.cgiar.org/ Table S5. Mean error (ME) and root mean square error (RMSE) using different global weather databases compared with local, high-quality control data during the growing season time period used in simulations of crop yields at each of four sites for rainfed maize in USA, irrigated rice in China, and rainfed wheat in Germany. [file gcb0019-3822-SD13.docx]

##### Supplementary information

Table S1-S3 Management parameters used in simulation models at 4 sites in 3 countries for 3 crops. Dates of planting, transplanting and physiological maturity are reported as day of the year (DOY). Maize crop variety expressed in relative maturity days (CRM).

Table S1

| Maize in USAA | | |
| --- | --- | --- |
| Sites | Planting date (DOY) | Variety (CRM) |
| Cedar Rapids (IA) | 120 | 110 |
| Grand Island (NE) | 124 | 106 |
| Lincoln (NE) | 119 | 114 |
| McCook (NE) | 126 | 110 |

**Table S2**

| Rice in China | | | | |
| --- | --- | --- | --- | --- |
| Sites | Planting date (DOY) | Transplanting date (DOY) | Variety name | Physiological maturity date (DOY) |
| Chengdu | 95 | 125 | 2You501 | 244 |
| Chongqing | 95 | 125 | 2You501 | 244 |
| Nanning, early season | 60 | 95 | IR72 | 201 |
| Nanning, late season | 196 | 217 | IR72 | 304 |
| Gushi, long season | 121 | 161 | XD90247 | 278 |
| Gushi, short season | 130 | 161 | 2You501 | 268 |

**Table S3**

| Wheat in Germany | | | |
| --- | --- | --- | --- |
| Sites | Planting date (DOY) | Variety name | Physiological maturity date (DOY) |
| Bad Herfeld | 279 | Tommi, 202 | 202 |
| Braunschweig | 278 | Tommi, 200 | 200 |
| Düsseldorf | 288 | Tommi, 199 | 199 |
| Leipzig-Schkeuditz | 278 | Tommi, 202 | 202 |

Table S4 Elevation within 100km of simulation sites in China (m). Source: CGIAR-CSI (2006): NASA Shuttle Radar Topographic Mission available for download at: <http://srtm.csi.cgiar.org/>

| **Site** | **Mean** | **Range** | **Standard Deviation** |
| --- | --- | --- | --- |
| Chengdu | 879 | 4746 | 819 |
| Chongqing | 449 | 2023 | 241 |
| Gushi | 105 | 1560 | 156 |
| Nanning | 180 | 1594 | 141 |

Table S5 Mean error (ME) and root mean square error (RMSE) using different global weather databases compared with local, high-quality control data during the growing season time period used in simulations of crop yields at each of four sites for rainfed maize in USA, irrigated rice in China and rainfed wheat in Germany.

|  |  | |  | |  | |  | |
| --- | --- | --- | --- | --- | --- | --- | --- | --- |
|  | Tmin (C°) | | Tmax (C°) | | Solar Radiation (MJ m^-2^) | | Water Deficit (mm) | |
|  | ME | RMSE | ME | RMSE | ME | RMSE | ME | RMSE |
|  | Maize | | | | | | | |
| NOAA | 0.40 | 1.33 | 0.95 | 1.34 | 17 | 126 | 34 | 131 |
| NCEP | 2.05 | 2.51 | 0.73 | 1.94 | 180 | 270 | -62 | 118 |
| CRU | -0.06 | 1.57 | 0.25 | 0.91 | -99 | 208 | 149 | 173 |
| NASA | 1.02 | 2.32 | -0.89 | 3.06 | 67 | 243 | 179 | 198 |
|  | Rice | | | | | | | |
| NOAA | -0.45 | 0.57 | -0.18 | 0.34 | 88 | 151 |  |  |
| NCEP | -3.39 | 6.57 | -3.82 | 6.39 | 895 | 2231 |  |  |
| CRU | -0.39 | 0.67 | -0.43 | 0.84 | 19 | 144 |  |  |
| NASA | -1.32 | 1.94 | -1.27 | 3.06 | 113 | 208 |  |  |
|  | Wheat | | | | | | | |
| NOAA | -0.56 | 1.55 | -0.16 | 2.37 | -29 | 75 | -15 | 50 |
| NCEP | -1.05 | 1.66 | -0.68 | 1.28 | 618 | 745 | -62 | 492 |
| CRU | -0.31 | 0.65 | -0.15 | 0.78 | -66 | 135 | 264 | 283 |
| NASA | -0.06 | 0.82 | -0.13 | 0.82 | -11 | 94 | 547 | 914 |
|  |  |  |  |  |  |  |  |  |
|  |  | | | | | | | |
